# Analysis of Clinical Outcomes in Pediatric Distal Tibia Triplanar Fractures Treated Surgically and Conservatively

**DOI:** 10.7759/cureus.20723

**Published:** 2021-12-26

**Authors:** Muhammet Salih Ayas, Muhammet Kalkışım, Mehmet Cenk Turgut, Recep Dincer, Oğuzhan Aslan, Kerim Öner, Ahmet Köse

**Affiliations:** 1 Department of Orthopedics and Traumatology, Karadeniz Technical University, Trabzon, TUR; 2 Department of Orthopedics and Traumatology, Sorgun State Hospital, Yozgat, TUR; 3 Department of Orthopedics and Traumatology, Erzurum Regional Training and Research Hospital, Erzurum, TUR; 4 Department of Orthopedics and Traumatology, Suleyman Demirel Universty Faculty of Medicine, Isparta, TUR

**Keywords:** aofas, earlier rehabilitation, conservative, surgical, triplane fracture

## Abstract

Purpose

We aimed to analyze the clinical and functional outcomes of patients who underwent surgery or received conservative treatment to look into the impact of treatment methods on clinical outcomes.

Methods

A retrospective study was performed on 25 patients with a minimum one-year follow-up. Patients were divided into two groups based on joint and physis displacement measured on preop CT images. Patients with a displacement of > 2 mm underwent surgery, while those with a displacement of < 2 mm received conservative treatment. The clinical results were assessed using the Ankle-Hindfoot Scale developed by the American Orthopedic Foot and Ankle Society (AOFAS) and the Modified Weber Protocol (MWP).

Results

The sample consisted of 14 patients who underwent surgery and 11 patients who received conservative treatment. The surgical group had a mean follow-up of 36.79±14.43 months, while the conservative group had a mean follow-up of 31.82±13.55 months. The surgical and conservative groups had a postop 1st-year AOFAS score of 96.64±3.54 and 93.64 ± 4.69, respectively. The difference was statistically insignificant (p > 0.05), but the surgical group had higher scores numerically. The surgical and conservative groups had a postop 6th-month AOFAS score of 84.64±1.64 and 80.82±2.85, respectively. The difference was statistically significant (p < 0.05).

Conclusion

The results of both surgical treatment and conservative treatment are satisfactory. Especially, surgical treatment should not be avoided in patients requiring surgery with a displacement of more than 2 mm and surgeons may consider surgery for better clinical outcomes and earlier rehabilitation in the treatment of triplane fractures.

## Introduction

Pediatric distal tibia triplane fractures are ankle injuries that affect skeletally immature children and account for 5% to 7% of all ankle fractures in that patient group [1]. Tillaux fractures always involve the ankle joint, but triplane fractures do not always extend to the ankle joint [2-4]. The distal tibial physis develops asymmetrically in children. Traumas in the pediatric age group are important because they affect the joint surface and pass through the growth cartilage [5-6]. If not treated properly, those fractures can cause physeal arrest, post-traumatic arthritis, chronic pain, and functional losses [7].

Physicians need more than standard anteroposterior (AP) and lateral radiography (LR) to diagnose triplane fractures and make surgical decisions [8]. Computed tomography (CT), in addition to AP and LR, helps physicians detect fracture configuration and inter-fragment displacement more accurately [9]. A more accurate diagnosis paves the way for better treatment options and reduces the risk of complications. There is still no consensus on surgical indication decisions for triplane fractures in the pediatric age group. Treatment decisions depend on the extent of displacement of the distal articular fracture, the displacement of the physis, or the presence of intra-articular displaced bodies. In case of a displacement of more than 2 mm on the articular surface, the rule of thumb is actually the necessity of anatomical reduction, but this indication has not yet been proven for the pediatric age group [10-13]. Another rule of thumb is checking the amount of displacement of the physis. Surgical reduction and fixation are recommended in the treatment of fractures with a displacement > 2-3 mm in the physis to reduce the premature closure of the distal tibia physis because the periosteum may interpose and affect healing [14-18]. However, there is no robust evidence to suggest that surgery can reduce the risk of the premature closure of the physis [19].

We used computed tomography to measure the displacement rate in pediatric distal tibia triplane fractures. We analyzed the clinical and functional outcomes of patients who underwent surgery or received conservative treatment in order to look into the impact of the treatment methods on clinical outcomes.

## Materials and methods

The sample consisted of 25 patients aged 8-16 years admitted to our center between January 2016 and January 2020 due to a triplane fracture of the distal tibia. Data were analyzed retrospectively. Informed consent was obtained from all participants. The study was approved by the Ethics Committee. The study was conducted according to the ethical principles outlined by the Declaration of Helsinki.

Participants either underwent surgery or received conservative treatment. Their clinical and functional outcomes were retrieved from the hospital registry system and documented. The inclusion criteria were at least one-year follow-up, preoperative CT imaging, adhering to controls, filling out the follow-up survey, and digitally accessible record. Exclusion criteria were a follow-up of less than one year and fractures around the ankle such as tarsal bone fractures, excluding fibula fractures.

Direct radiographs, CT scans, clinical notes, and technical data (open reduction + internal fixation and closed reduction + plastering) and complications (loss of reduction, malunion, implant failure, additional procedure, deep or superficial infection, revision surgery, and leg length discrepancy due to premature physis closure) were recorded. A discrepancy of > 1 cm on radiography and tape measurement was defined as a leg length discrepancy (LLD), while a deformity of > 5 ° on anteroposterior and lateral radiographs was defined as angular deformity [3].

Closed reduction and splint were applied to all patients included in the study after direct radiographic examination in the emergency department, especially for those with large displacements. It was then evaluated with CT imaging. Treatment decisions were based on CT images after closed reduction. Displacement was measured using the farthest axial distance. Patients were divided into two groups based on joint and physis displacement, the distance between bone fragments measured on preop CT images. Patients with distance and displacement of > 2 mm underwent surgery, while those with a displacement of < 2 mm received conservative treatment [1,19]. The surgical group was treated with open reduction + internal fixation, while the conservative group was treated with closed reduction + casting.

Surgical technique

Surgery was performed using a pneumatic tourniquet control applied to the limb of the injured side. Anterolateral, anteromedial, and/or posterolateral incisions were used depending on the configuration of the fractures. The nerves in the incision site were explored and preserved. After reaching the joint, it was washed to remove mini joint fragments and the fractured hematoma. Cartilage structures were examined to identify additional injuries. Periosteum and soft tissues were removed. C-arm radiographic fluoroscopy was used to determine the ideal screw length, to confirm the location of the screw, and to avoid damaging the physis and screwing through the physis. Internal fixation was achieved after anatomical reduction. Fixation was performed from the inferior and superior of the physis parallel to it but without passing through its line. After surgery, the ankle was splinted in a neutral position.

Conservative treatment - casting technique

When the conservative group patients were admitted to the emergency or orthopedic outpatient clinic for the first time, they underwent closed reduction and long leg casting with the ankle in a neutral position.

The conservative group patients with long leg casts were kept immobile for six weeks, while the surgical group patients with short leg casts were kept immobile for four weeks. After cast-splint fixation, all patients were allowed to do active and passive range of motion exercises and put as much weight on their feet as they could tolerate. After eight weeks, all patients placed full weight on their feet and continued undergoing rehabilitation for four more weeks under the supervision of a physical therapist.

Radiological evaluation

**Figure 1:**
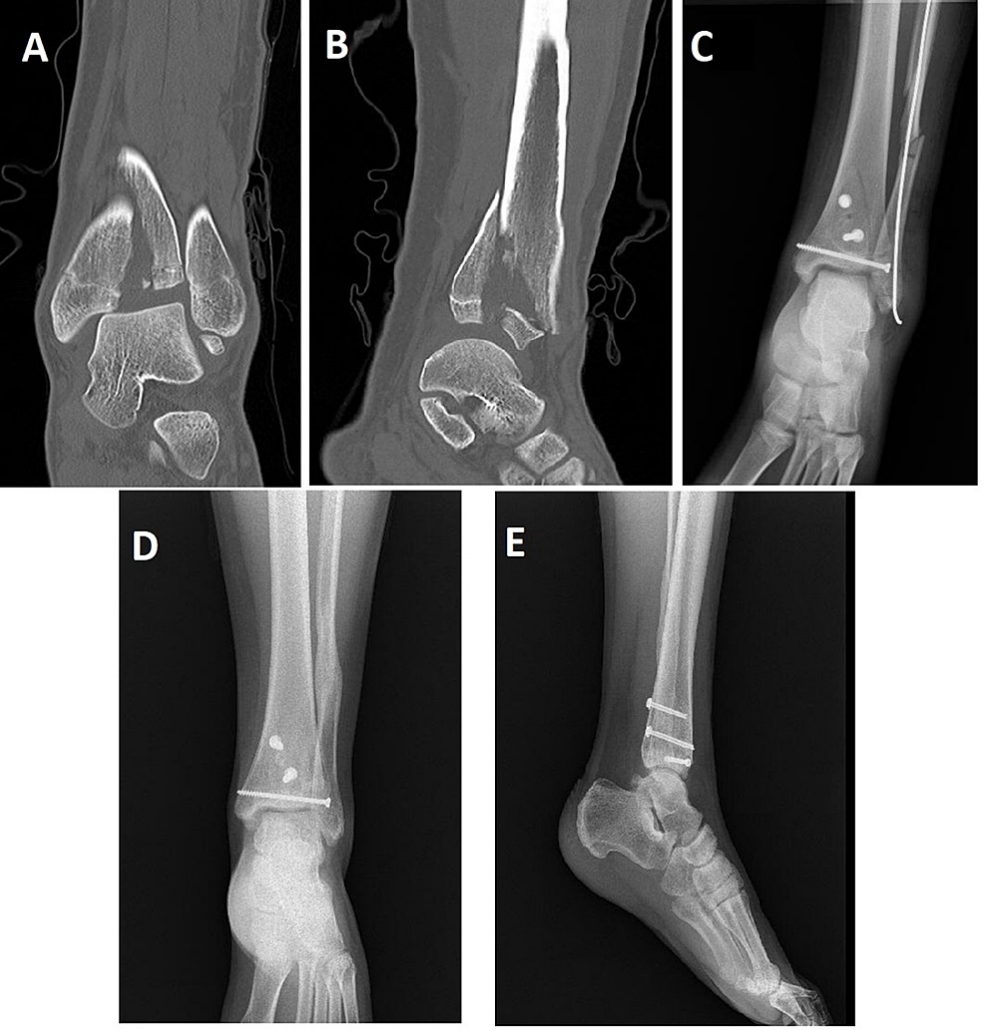
Radiological Evaluation - Preoperative, postoperative, and follow-up imaging of the left ankle triplane fracture A: Preoperative sagittal CT image of the ankle showing the displacement and configuration of the fracture; B: Preoperative coronal CT image of the ankle showing the displacement and configuration of the fracture; C: Anteroposterior radiograph of the ankle showing the postoperative anatomical reduction and fixation, as well as the position of the implants that have never passed through the physics; D: Anteroposterior radiography of the ankle showing the radiological union of the fracture in clinical follow-ups; E: Lateral radiography of the ankle showing the radiological union of the fracture in clinical follow-ups.

All patients were assessed using direct radiography two, four, six, eight, 12, and 24 weeks and one year after surgery and at the last follow-up control (Figure 1). 

Postoperative pain and clinical evaluation

Postoperative pain was assessed before surgery and six and 12 months after surgery and at the last clinical control using Wong-Baker FACES® Pain Rating Scale [20-21]. The instrument was developed to help children communicate about their pain. Today, it is widely used all over the world for children aged three or older. It facilitates communication and improves pain assessment. The two groups were analyzed. it was explained to the patients that each face represents a person who has no pain (hurt), or some, or a lot of pain. Face 0 doesn’t hurt at all. Face 2 hurts just a little bit. Face 4 hurts a little bit more. Face 6 hurts even more. Face 8 hurt a whole lot. Face 10 hurts as much as you can imagine, although you don’t have to be crying to have this worst pain. The patients were asked to choose the face that best depicts the pain they are experiencing [22].

Clinical results were assessed six and 12 months after surgery and at the last clinical examination. The clinical results were assessed using the Ankle-Hindfoot Scale developed by the American Orthopedic Foot and Ankle Society (AOFAS) [23-24] and the Modified Weber Protocol (MWP) [25-26].

The AOFAS assesses pain (40 points), function (50 points), and alignment (10 points). It is based on subjective and objective findings incorporated into a numerical score [23-24].

The Modified Weber Protocol assesses the function of the talocrural joint, walking ability, and radiographic results on a scale of one to four. The total score is the sum of the scores of those three categories (5= excellent; 6= good; 7 = fair; > 7 = poor) [25-26].

Statistical analysis

The data were analyzed using the Statistical Package for Social Sciences (IBM Corp. Released 2015. IBM SPSS Statistics for Windows, Version 23.0. Armonk, NY: IBM Corp.) at a significance level of < 0.05. Mean and standard deviation was used for descriptive statistics, while numbers and percentages were used for categorical statistics. Histograms were used for normality testing. The results suggested non-normal distribution. Therefore, nonparametric tests (Mann-Whitney U test. Chi-square, and Fisher's Exact test) were used to compare the groups.

## Results

The sample consisted of 14 patients who underwent surgery (surgical group) and 11 patients who received conservative treatment (conservative group). The surgical group had a mean follow-up of 36.79±14.43 months, while the conservative group had a mean follow-up of 31.82±13.55 months. Their data were analyzed retrospectively (Table 1).

**Table 1:** Demographic data of the patients

Features		Surgical group (n=14)	Conservative group (n=11)	p
Age (Mean±SD)		11.50±2.27	11.73±2.00	0.719
Follow up (months)		36.79±14.43	31.82±13.55	
Gender	Male	9	7	0.973
	Female	5	4	
Side	Right	7	6	0.821
	Left	7	5	
Number of Fragments	2	10	11	0.053
	3	4	0	
Fracture configuration	Lateral triplane	6	8	0.135
	Medial triplane	8	3	
Trauma	Skiing	3	0	0.052
	Slip Down	1	4	
	Soccer	1	0	
	Sports Activity	1	4	
	Traffıc Accıdent	6	1	
	Fall Down	2	2	

Table 2-3 shows the patients’ postop clinical evaluation scores. The surgical group had significantly different six-month postop MWP and AOFAS scores (p < 0.05). However, there was no significant difference between one-year and last-control MWP and AOFAS scores. On the other hand, the surgical group had a higher-excellent rate in the first year.

**Table 2:** Postoperative Evaluation of Modified Weber Protocol Scores.

		Surgical group (n=14)	Conservative group (n=11)	p
Postoperative 6th month	Excellent	0	0	0.011
	Good	0	0	
	Fair	12	4	
	Poor	2	7	
Postoperative 1st year	Excellent	8	3	0.320
	Good	5	7	
	Fair	1	1	
	Poor	0	0	
Postoperative final	Excellent	14	10	0.250
	Good	0	1	
	Fair	0	0	
	Poor	0	0	

**Table 3:** Evaluation of Clinical Scores Between Surgical and Conservative Groups. AOFAS Score: American Orthopedic Foot and Ankle Society Score

	Surgical group (n=14)	Conservative group (n=11)	
	Ort±SS	Ort±SS	p
Wong-baker faces (Preoperatıve)	7.29±0.99	5.64±1.50	0.007
Wong-baker faces (Postoperative 6th month)	1.00±1.03	2.00±1.54	0.097
Wong-baker faces (Postoperative 1st year)	0.14±0.53	0.18±0.60	0.861
AOFAS Score (Postoperative 6th month)	84.64±1.64	80.82±2.85	0.001
AOFAS Score (Postoperative 1st year)	96.64±3.54	93.64±4.69	0.068
AOFAS Score (Postoperative final)	99.57±1.08	99.27±2.41	0.771

Superficial infection was observed in one surgical group patient, who was hospitalized and treated with IV antibiotherapy. No surgical debridement was performed. Other patients in the surgical group did not develop any complications (loss of reduction, malunion, implant failure, additional procedure, deep or superficial infection, revision surgery, and leg length discrepancy due to premature physis closure). During the follow-up period in the surgical group, implants were not removed in any of the patients and it was not considered necessary. No complication was observed in the conservative group.

## Discussion

We used the MWP and AOFAS scores to compare the treatment outcomes of surgery and conservative treatment for distal tibia triplane fractures. The surgical and conservative groups had a postop first-year AOFAS score of 96.64±3.54 and 93.64 ± 4.69, respectively. The difference was statistically insignificant (p > 0.05), but the surgical group had higher scores numerically. The surgical and conservative groups had a postop sixth-month AOFAS score of 84.64±1.64 and 80.82±2.85, respectively. The difference was statistically significant (p < 0.05).

Distal tibia triplane fractures are intra-articular fractures that may include the physis. Even if those fractures are identified through standard radiographs, it is not enough to make a surgical decision. In a retrospective study in 2020, Lurie et al. reported that functional results were similar to surgical treatment when the intra-articular gap did not exceed 2.5 mm. The researchers concluded that surgery should be the treatment option for displacements exceeding 2.5 mm [3]. Computed tomography is required to make this precise measurement and identify the displacement in the joint and physis [14]. Physicians should use CT to evaluate patients with triplane fractures preoperatively to achieve better treatment outcomes.

Conservative treatment for triplane fractures may yield positive outcomes. However, a displaced triplane fracture may cause LLD and/or degenerative osteoarthritis of the joint due to physeal arrest because it may involve the physis and then extend into the joint [27,28]. The joint surface and the physis should be anatomically reduced and fixed to minimize the risk of complications [14-18]. We also agree with this view and we planned the treatment of our patients accordingly. All our patients in the surgical group had excellent AOFAS and MWP scores. Only one of them developed a superficial infection, whereas none developed physis arrest and LLD.

El-Karef et al. ​​​​​performed open surgery on 21 patients with triplane fractures. They followed up with their patients for an average of 28 months and used a clinical follow-up score they developed to evaluate them. They classified fourteen patients as excellent, four as good, and one as fair. Three patients had restricted ankle movements by less than 15°, while four had a mildly reduced subtalar arc of movement. They reported that the radiographs of two ankles showed varus deformity of the distal tibia less than 5°. Three patients presented with valgus deformity of the distal tibia in the range of 3° to 7°. Radiologically, three patients had an LLD of less than 5 mm, while two had an LLD of 5 to 10 mm [14]. We detected no complications in the surgical group, except one case of superficial infection. The reason for this is probably; anatomically reducing the physis line and avoiding screwing through the physis. We think that following these steps results in fewer complications.

There is little research on the health outcomes of surgery and conservative treatment for distal tibia triplane fractures. Lurie et al. followed up 57 patients with triplane and tillaux fractures for at least two years. They clinically evaluated Foot and Ankle Ability Measure (FAAM) Sports and Single Assessment Numerical Evaluation (SANE) Sports scores and found no statistical difference between the surgical and conservative groups. They reported nine complications (superficial wound infection, regional pain syndrome, keloid formation, premature physeal closure, and osteolysis around a screw) in the surgical group and only one complication (premature physeal closure) in the conservative group [3]. We observed no complications, except one (superficial wound infection) in the surgical group.

Seung Min Ryu et al. compared the treatment outcomes in patients with triplane fractures who were treated conservatively (n=19) or surgically (n=14) and followed them up for two years. The researchers did not find any significant difference in AOFAS and MWP clinical scores between the two groups. They reported LLD in one patient in each group. Therefore, they have concluded that conservative treatment and surgery have similar clinical and radiographic outcomes but that conservative treatment is a better option than surgery because the former does not have the risks that come with the latter [26]. It was the most similar study to ours regarding the number of patients and mean follow-up time. We observed no LLD in our patients and no significant difference between 1st-year and last-control AOFAS and MWP scores. We also think that displacements < 2mm have acceptable outcomes [18]. However, surgical treatment should not be avoided in patients with displacement greater than 2 mm.

Limitations

The study had two limitations. First, it was retrospective. However, we work in a trauma clinic where we keep the data of all our patients regularly and systematically. Second, the sample size was not very large. However, we think that it was large enough given the fact that triplane fractures are rare injuries. Prospective randomized controlled trials with larger samples are warranted to obtain better results.

## Conclusions

The results of both surgical treatment and conservative treatment are satisfactory. This article can be considered valuable as there are not many studies on this subject. Although the comparison is not appropriate, It shows that surgical treatment is an effective and reliable method and has few complications. Surgical outcome with appropriate indication is satisfactory and surgeons should not be worry when deciding on surgery. It may considered surgery for better clinical outcomes and earlier rehabilitation in the treatment of triplane fractures.
